# Medico-Legal Implications of Video Recordings of Clinic Visits in Malpractice Claims Against Medical Providers

**DOI:** 10.7759/cureus.53627

**Published:** 2024-02-05

**Authors:** Komal Naeem, Malika Bhargava, Randall W Porter

**Affiliations:** 1 Department of Neurosurgery, Barrow Neurological Institute, Phoenix, USA

**Keywords:** patient-provider communication, neurosurgery, medico-legal implications, malpractice claims, audio-video recording of clinic visits

## Abstract

Objective: Although audio-video recordings of clinic visits improve patient satisfaction and recall, the associated presumed risk of increased malpractice claims limits their use. In this study, we identified whether video recording clinic visits was associated with increases in professional liability claims.

Methods: From 2015 to 2017, the institution’s loss run was analyzed, and the rates of medical malpractice claims per physician-year were compared between physicians who used video recordings of clinic visits (V-RoCs) and those who did not. The term “users” was applied to all physicians whose mean percentage of patient visits with video recording was greater than the mean percentage for the practice overall.

Results: Over three years, 15,254 patients used V-RoCs. The use of video recordings for clinic visits increased at a rate of 23% per year. No association was found between video recordings and increased malpractice claims. The rate of all claims between users and nonusers did not differ significantly (P=0.66). Of seven paid claims or lawsuits from 2000 to 2017, none were against physicians who used video recordings.

Conclusion: Video recording of patient-physician encounters was not associated with an increase in malpractice lawsuits. According to federal law, a patient can legally record a clinic encounter without physician consent, which has many ethical implications. Formalizing the recording process is beneficial for both parties and allows the resource to be used to its maximum potential.

## Introduction

Recent advances in technology, including the video recording of patient-physician communication and telemedicine, are revolutionizing health care provision. Such technology has become more important since the onset of the coronavirus pandemic in the United States in early 2020 when many patients and their attendants began to have limited access to medical facilities.

The popularity of recording clinic visits is evident from a survey conducted in the United Kingdom that included 130 participants. The desire to record a clinic visit, with or without the physician’s permission, was reported by 69% of the participants, whereas 15% admitted to secretly recording their visits. The fear of physician refusal, which could potentially harm the patient-physician relationship, was a common reason for surreptitious recording [1]. In 38 US states (76%), it is legal to record a conversation as long as at least one party consents; hence, a patient is not legally liable for covert recording. However, this has several ethical implications, such as the possibility of the physician feeling betrayed and terminating the patient-physician relationship and the risk of breaching patient confidentiality [2,3]. As of February 2019, 81% of Americans own a smartphone [4]. Because recording technology is easily accessible, it is difficult to prevent surreptitious recording [1]. Therefore, it is believed that formalized recording by health care providers can make the process more organized and minimize the risk of breaching patient privacy [2].

For patients with complex diseases, high morbidity, or the inability to retain all the information imparted by health care providers, participation in decision-making regarding their treatment can be hindered. The merits of providing patients with their visit summary in the form of a document, audiotape, or video recording have been studied widely among oncology patients. The efficacy of this intervention was established by several studies, including randomized controlled trials, with the conclusion that providing patients with a visit summary in the form of a document, audiotape, or video recording improves patient understanding and recall, consequently helping patients with decision-making [5-17]. In our practice, the senior author (RWP) introduced a smart-device application system (The Medical Memory, LLC, Phoenix, AZ) for the video recording of clinic visits (V-RoC) in 2015. Despite the proven efficacy of this intervention, concerns regarding the presumed unfavorable medico-legal consequences of V-RoC have made physicians hesitant to use it. The neurosurgery setting further exacerbates this concern, as neurosurgery has one of the highest mean malpractice payments and the highest rate of recurring claims among US physician specialties [13,18-21]. It is estimated that by 65 years of age, 99% of physicians in specialties associated with a high risk of litigation (including neurosurgery) will have faced a medical malpractice claim, and 65% will have had an indemnity payment [20]. Because this presumed concern can potentially limit the use of V-RoC technology, it is important to analyze the effect of V-RoC on rates of malpractice claims. In this study, we evaluated the frequency of the occurrences of claims against the physicians in our practice and analyzed whether the introduction of V-RoC affects the rate of these claims.

Parts of this paper were presented at the Congress of Neurological Surgeons Annual Meeting 2019 (October 21, 2019; San Francisco, CA), the 17th International Conference on Communication in Healthcare (October 26, 2019; San Diego, CA) and the 2020 American Association of Neurological Surgeons Annual Scientific Meeting, E-presentation.

## Materials and methods

**Figure 1:**
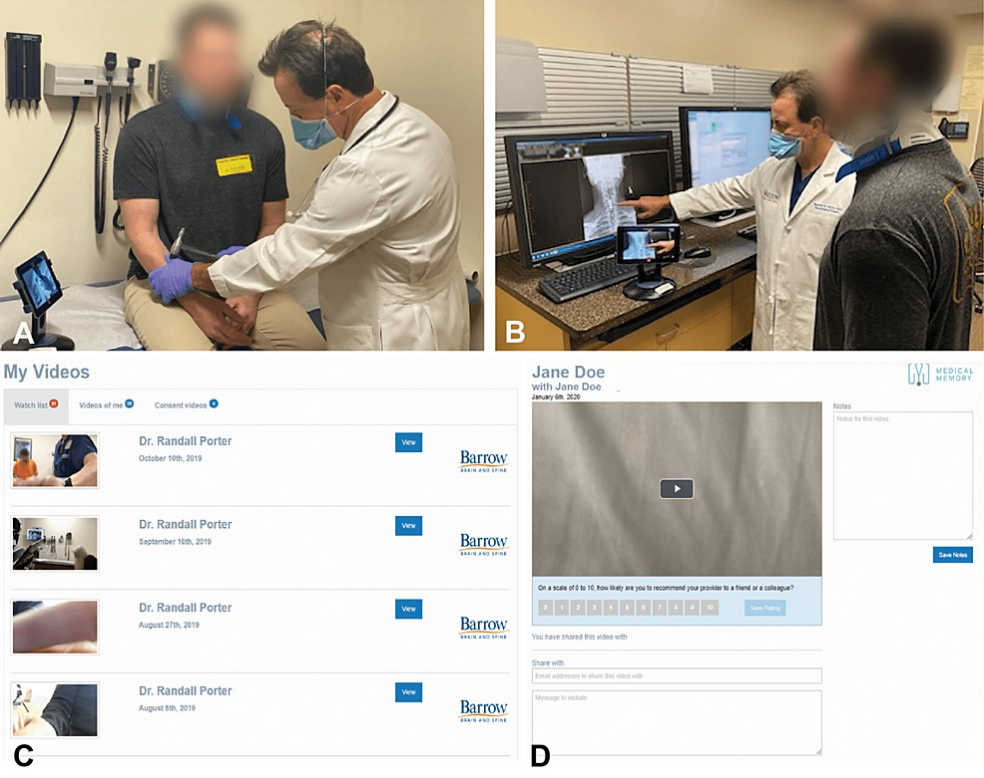
Setup for the video recording of a clinic visit and screenshots of the application used in this study (The Medical Memory, Phoenix, AZ) (A) A tablet with a built-in microphone and camera is placed on the table for recording the patient-physician communication. (B) The tablet is placed on a stand so that it can be moved easily. In this image, the physician is recording while explaining the patient’s disease process on the radiological image. (C) A screenshot of the patient’s view of the application. All recorded videos are listed with the providers’ names and the dates and locations of the recordings. Patients are notified when their video gets uploaded to a Health Insurance Portability and Accountability Act–compliant server. (D) A screenshot of the patient’s view while watching the video, with the feedback survey link below the video. The sharing option can also be seen at the bottom of the screen. The video can be shared by entering an email address and an optional message. The patient can share videos with anyone they choose (including family, friends, physicians, nurse practitioners, and other health care providers). However, shared videos cannot be shared again by the recipient, nor can they be downloaded from or uploaded to the Internet (including social media). A text box for notes is provided in the right corner of the screen. Used with permission from Barrow Neurological Institute, Phoenix, Arizona

Video recording was introduced in our practice in 2009, but it remained sporadic until 2015 when a smart-device application system (The Medical Memory) was launched for recording and sharing videos, and a smart tablet with a built-in camera and microphone replaced the digital camera (Video 1; Figure 1).

**Video 1:** An edited video of a preoperative visit Informed patient consent was obtained for the use of this video. Used with permission from Barrow Neurological Institute, Phoenix, Arizona.

A Health Insurance Portability and Accountability Act of 1996-compliant server is used to upload, store, view, and share the videos, which ensures privacy via an encrypted connection. Videos are not stored on the tablet, which prevents the misuse of the videos in case of theft or loss of the device. Additionally, a global positioning system tracker notifies the company if a tablet leaves the clinic.

Each patient visiting our clinic may opt for a V-RoC without any additional fee. Upon patient agreement, an electronic consent form is signed. Patients are notified that these videos will not become a part of their electronic medical records. The video can be paused once the recording has started if they feel uncomfortable discussing their concerns openly. Patients are also informed that their refusal will not affect their relationship with the physician. The tablet is placed on a stand (so that it can be moved around) in the corner of the room, facing both the patient and the physician so that both are in the frame, but in such a way that the tablet does not intervene in or restrict the interaction (Figures 1A, 1B). Later, patients receive a notification when their video is uploaded, and they can log in using a username and password provided earlier. Patients can view the list of all videos (in case of multiple visits) and watch and share them. A shared video cannot then be shared again by its recipient, which provides more security (Figures 1C, 1D). Medical providers (including physicians, nurse practitioners, and physician assistants) are also provided with a login and may revisit their patients’ videos. These videos are stored indefinitely.

We performed an in-depth analysis of our institution’s loss run. We queried all closed claims against the physicians practicing at our high-volume center (Barrow Neurological Institute, Phoenix, AZ) from 2000 to 2017. We included all professional liability claims, including precautionary claims (in which the physician “red-flagged” the case due to an adverse outcome, but no formal complaint was filed), claim actions (in which a patient filed a complaint demanding compensation, usually monetary), and lawsuits (when the case goes to trial in the courtroom). “Users” were defined as physicians whose three-year mean percentage of patient visits with V-RoC use was greater than the three-year mean percentage for the practice overall for 2015 through 2017. To assess the effect of V-RoC use on claim rates, rates of claims per physician-year (calculated by dividing the number of physicians who had claims [claim actions or lawsuits] within a year by the number of physicians who practiced in that year) were compared between V-RoC users and nonusers [19].

Ethical approval

This is a retrospective review of de-identified data; therefore, the requirement of informed consent was waived.

Statistical analysis

Data analysis was performed using Microsoft Excel (Microsoft Corporation, Redmond, WA). A t-test was used to compare continuous variables.

## Results

**Figure 2:**
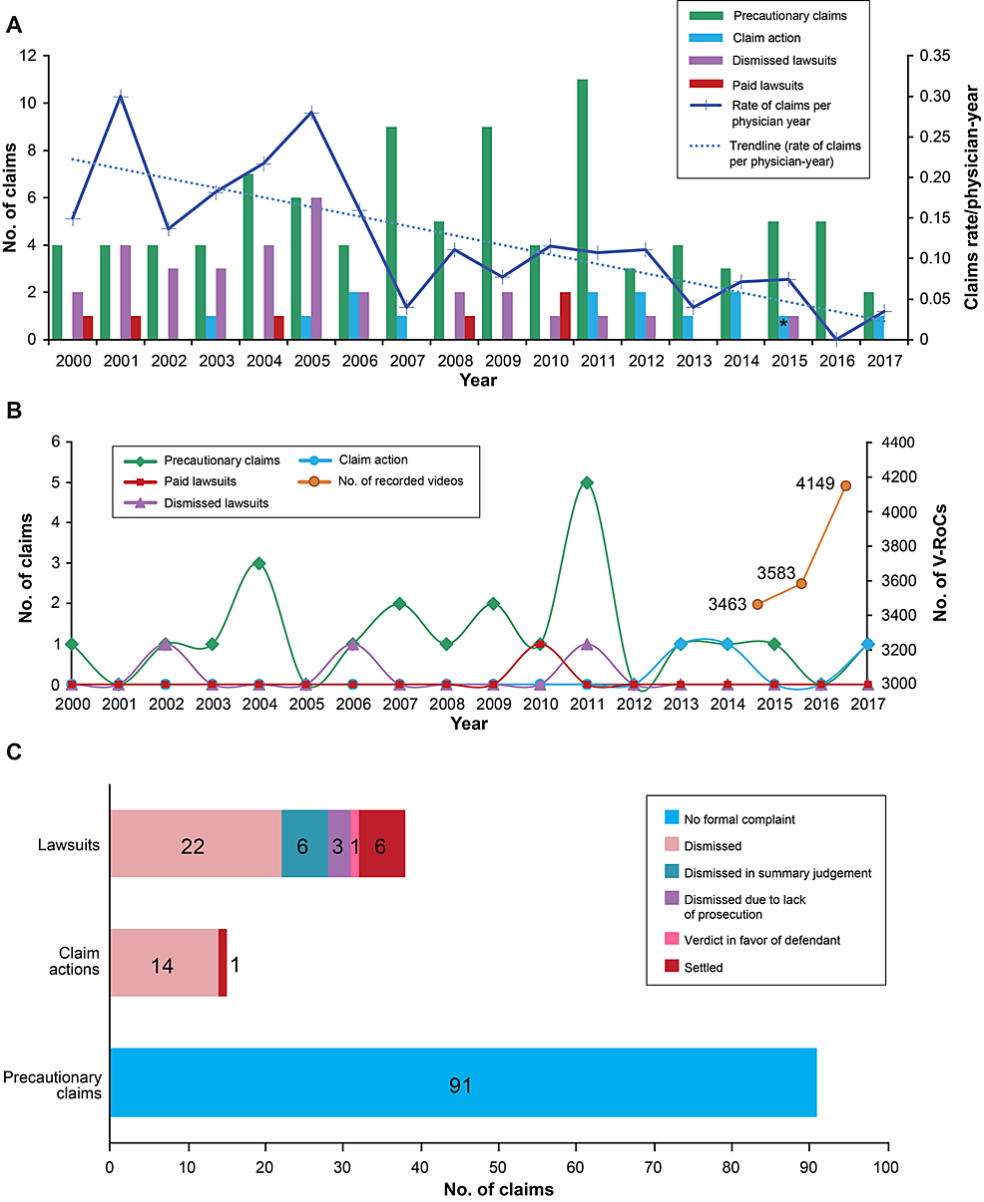
The trend of claims from 2000 to 2017 (A) Frequency of precautionary claims, claim actions (dismissed and paid), and lawsuits (dismissed and paid) against all neurosurgeons working at our institution. The rate of claims per physician-year showed a downward trend. The asterisk indicates a claim action settled against a nonuser in 2015. (B) Claims against four physicians (defined as “users”) whose three-year mean percentage of patient visits with the use of video recording of clinic visits (V-RoC) was greater than the three-year mean percentage for the institution as a whole, along with the number of videos recorded by users during the period 2015 to 2017. (C) Bar graph illustrating the distribution of precautionary claims, claim actions, and lawsuits against all physicians from 2000 to 2017. Used with permission from Barrow Neurological Institute, Phoenix, Arizona.

Claims against all physicians in the practice trended downward from 2000 to 2017 (Figure 2A).

Of 98,474 patients, a total of 15,254 (15%) used V-RoCs from 2015 to 2017. Although the annual increase in V-RoC use was 9%, no claims were filed by the patients using it.

All physicians were divided into two groups, depending on their percentage of V-RoC use. The rate of medical malpractice claims per physician-year between V-RoC users and nonusers was not significantly different (mean difference, 0.04; 95% CI, -0.21 to 0.28; P=0.66) for all claims (Table 1).

**Table 1:** Comparison of the rate of medical malpractice claims per physician-year between physicians who used video recordings of clinic visits (users) and those who did not (nonusers) CI, confidence interval Users are defined as those physicians whose mean percentage of patient visits with video recordings was greater than the mean percentage for the practice overall. ^a^P=0.66

Parameters	No. of patients	No. of physicians	Rate of medical malpractice claims per physician-year
Users			
2015	3845	4	0
2016	5575	4	0
2017	5834	4	0.25
Nonusers			
2015	27,019	23	0.09
2016	27,830	24	0
2017	28,371	25	0.04
Difference between users and nonusers, 2015-2017, mean (95% CI)			0.04 (-0.21 to 0.28)^a^

In our physician cohort, four V-RoC users were identified who met the criteria. All of them had established practices before the introduction of V-RoC. We identified two precautionary claims and one dismissed claim action against the V-RoC users from 2015 to 2017, compared with 15 precautionary claims, two dismissed claim actions, two dismissed lawsuits, and one paid lawsuit from 2000 to 2014 (Figure 2B).

We identified 144 occurrences in our institution’s loss run from 2000 to 2017 against all physicians. Precautionary claims accounted for almost two-thirds of all occurrences (91/144, 63.2%), and 53 (37%) claims were formally filed. In total, seven (4.9%) claims (six lawsuits and one claim action) were paid or settled (no catastrophic payment). All but one of the claim actions were closed without any payments. Approximately four-fifths (32/38, 84%) of the lawsuits filed were dismissed or resulted in a verdict in favor of the defendant (Figure 2C).

## Discussion

Several insights regarding professional liability were garnered from our database. More than 15,000 patients have opted to use V-RoC since its introduction in our clinic, and none of these patients has filed a paid claim or lawsuit against the physician. We found no difference in the rate of malpractice claims per physician-year between V-RoC users and nonusers (P=0.66). Tracking claims against users revealed no increase in the frequency of precautionary claims, indicating that V-RoC use does not increase anxiety regarding litigation among physicians. This is a preliminary study aimed at identifying the patterns of malpractice claims associated with the use of V-RoC. It can be argued that there is a potential selection bias because physicians with good communication skills and experience are possibly more likely to use video recording. Establishing causality and identifying confounding factors are difficult in this retrospective, nonrandomized study.

A study reported that only 19% of filed claims from 1991 to 2005 resulted in an indemnity payment in neurosurgery [20]. Similarly, we found that seven of 53 (13.2%) claims were settled. These data raise concern that the assumed burden of professional liability is greater than the actual burden, which may hinder the provision of efficient and improved health care. Due to the presumed increased risk of professional liability associated with neurosurgery and video recording, the platform used at our institution for the video recording provides an additional coverage of $1,000,000 in case of a lawsuit.

Physicians who allow themselves to be recorded increase transparency, strengthening the trust between the patient and the physician. Notably, in one instance, a patient filed a malpractice lawsuit because of an intraoperative complication without mentioning the name of the attending physician (who was a user), even though that physician was the primary surgeon (this case was, therefore, not included in the analysis). It is postulated that the strengthening of the physician-patient relationship by video recording can serve as one of the factors contributing to this patient’s behavior. Moreover, physicians can also see the use of V-RoC as an opportunity to improve their communication skills because the awareness of being recorded may encourage a conscious effort to be thorough [2]. The video recordings of the clinical encounters can also serve as courtroom evidence in malpractice cases. This objective record may benefit physicians because juries typically give more credence to a patient’s account of events; it is popularly believed that a patient’s memory of their unique clinical visit is more reliable than that of a physician who sees multiple patients with similar diagnoses in a single day [10].

For this group analysis, it is important to consider a few assumptions. V-RoC is a relatively new concept, as is evident from its use. During the study period, the proportion of V-RoC users increased every year. This type of increase is usually seen when a new technology is released, as usage increases over time. Therefore, in this study, a dichotomous division of users and nonusers among physicians was not possible. Additionally, patients were voluntarily choosing to have recordings made. Thus, it was unlikely that any physician using V-RoC had all patients opting for the recording and vice versa. Hence, we decided to use the mean rate of use as a cutoff. Therefore, while comparing the mean physician-claim rate, it is important to keep in mind that those physicians had a mix of patients using V-RoC. Nonetheless, we do not report any occurrence of a paid/settled claim filed by a patient who opted for V-RoC from 2015 to 2017.

Future plans

V-RoC has tremendous potential benefits that will be revealed as its use becomes more widespread. The use of V-RoC among hospitalized patients is currently being studied. In that setting, patient understanding may be hampered by altered mental status, drowsiness during doctor visits, and changing attendants. Researchers can use these videos to study different aspects of physician-patient communication, including nonverbal components, and collect data regarding details that are often missed in the electronic medical record. V-RoC can also replace photographs to record patient progress, such as improvement in facial nerve functioning, limb strength, and gait. Furthermore, it provides the added advantage of allowing patients to review their progress. Lastly, the use of V-RoC with artificial intelligence and natural language processing in medicine is also being explored.

Limitations

In this study, only three-year data for users could be analyzed because of a two-year statute of limitations for malpractice claims; therefore, a meaningful comparison between V-RoC users and nonusers was not possible. Analysis of long-term data will resolve these issues and assist in making stronger inferences. There are many potential confounders, as discussed above, and the data for the number of claims is small, which makes it difficult to adjust for confounders. This is a preliminary study that provides an initial report on the rate of malpractice claims among physicians using V-RoC. A future study with a larger sample size and a detailed description of when physicians and patients accept or refuse the use of this technology will provide a more comprehensive data set that can be used to calculate the adjusted rates.

## Conclusions

In conclusion, the audio-video recording of clinic visits was not associated with an increase in malpractice claims or litigations. We report no paid claims or lawsuits during a three-year period among 15,254 patients who opted to have their clinic visits recorded. Formalized audio-video recordings of patient clinic visits can change how health care information is provided to patients, increase their understanding, and improve their recall of information.
